# Dual-Layer PVA-HNT/PTFE Membranes for Boosted Antiwettability and Stability in Membrane Distillation

**DOI:** 10.3390/membranes16060201

**Published:** 2026-06-09

**Authors:** Guang Yang, Yu Song, Xianghe Kong, Zi Yang, Qing Chen, Hang Xu

**Affiliations:** 1 College of Environmental Science, Hohai University, Nanjing 210098, China; guang.yang@sylu.edu.cn; 2 Suzhou Litree Ultra-Filtration Membrane Technology Co., Ltd., Suzhou 215000, China; 3 School of Environmental and Chemical Engineering, Shenyang Ligong University, Shenyang 110159, China

**Keywords:** poly (vinyl alcohol), halloysite nanotube, antiwetting, membrane distillation

## Abstract

Separation membranes with inherent antiwettability and stability are highly desirable for membrane distillation (MD) in practical applications. In this study, hydrophilic–hydrophobic dual-layer membranes composed of a dense poly (vinyl alcohol)/halloysite nanotube (PVA-HNT) layer and a microporous polytetrafluoroethylene (PTFE) layer were fabricated to improve wetting and fouling resistance during the MD process. The incorporation of the HNT manipulated the crystallization and chain mobility of PVA, endowing the PVA-HNT layer with tunable water transport properties by adjusting the level of HNT loading. Benefiting from the hydrophilic top layer on PTFE, the dual-layer membrane with an optimal HNT loading of 5 wt% showed stable water vapor flux (7.6 kg/m^2^·h) while maintaining salt rejection above 99.95%. This performance was achieved using a 3.5 wt% NaCl feed solution with 0.4 mM sodium dodecyl sulfate at a feed temperature of 50 °C and permeate temperature of 10 °C. In contrast, the pristine PTFE membrane suffered from severe pore wetting, with its salt selectivity dropping from 99.5% to 91.5%. Antifouling performance was further evaluated using real landfill leachate in a 50 h treatment. The dual-layer membrane with a 5 wt% HNT maintained stable separation behavior with a 15.3% decrease in water flux, whereas the flux of the PTFE membrane declined by 70.5% in 30 h of operation. A distinct fouling layer was observed on the PTFE membrane surface after the operation, while no obvious fouling was identified on the dual-layer membrane, confirming its superior antifouling properties.

## 1. Introduction

Separation by membrane distillation (MD) is a well-established liquid mixture processing technology realized via the selective transport of vapor through a hydrophobic microporous membrane, while the liquid, such as water, and dissolved contaminants are effectively repelled. The fundamental driving force for mass transfer in this scenario is the vapor pressure difference across the membrane due to the presence of a temperature gradient between the hot feed stream and the cold permeate side [1,2]. Amid rising demand for sustainable development and resource recovery, MD technology has gained increasing research attention for the separation of volatile and nonvolatile substances as well as wastewater treatment [3,4,5]. Compared with reverse osmosis and nanofiltration [6,7], MD operates under near-atmospheric pressure without requiring high hydraulic pressure on the feed side. It also exhibits superior tolerance to highly concentrated feeds and enables the almost complete removal of nonvolatile molecules including salts and heavy metals. Moreover, when waste heat or renewable thermal energy is accessible, the permeate flux of MD can be flexibly regulated by adjusting the temperature gradient, which helps to reduce reliance on external energy input and lower the overall energy consumption of the separation system [8,9,10].

To date, membrane wetting and fouling constitute the major bottlenecks impeding the large-scale industrial deployment of MD [11,12,13,14]. During MD operation, hydrophobic membranes are vulnerable to wetting and fouling phenomena due to the presence of organics and surfactants such as humic acid (HA), sodium dodecyl sulfate (SDS), Tween 20, etc., which consequently lead to a decline in separation performance [15]. Wang et al. conducted a comprehensive study on the wetting characteristics of a polyvinylidene fluoride (PVDF) membrane influenced by various types of wetting agents (ethanol and Triton X-100) in the direct contact membrane distillation (DCMD) process [16]. The results revealed that wetting occurred instantaneously with the gradual and dynamic evolution of water flux, leading to a decline in the overall separation performance of the PVDF membrane. So far, hydrophilic modifications have been employed to render chemical and structural changes in membranes, exhibiting significant enhancements in antiwetting and antifouling properties [17,18,19,20]. The grafting of hydrophilic moieties onto MD membrane surfaces may compromise the liquid entry pressure (LEP) and exacerbate the risk of membrane wetting, should these functionalities infiltrate into the membrane pores. On the other hand, coating a hydrophilic and permeable layer to avoid contact with wetting agents or fouling components while preserving the LEP is preferable. In order to enable a high-performance MD process without a significant sacrifice of membrane throughput, coating materials with inherent mass transfer features are required [12,21,22,23].

In recent years, halloysite nanotubes (HNTs) have emerged as a promising nano-scale functional material for membrane separation applications owing to their unique structural features, exceptional mechanical properties, and large specific surface area [24,25,26]. HNTs are a type of natural silicate mineral with a hollow tubular structure and surface -OH functional groups. The hydrophilic nature of HNTs makes them particularly attractive for the hydrophilic modification of MD membranes. Compared to conventional nanoparticles such as carbon nanotubes and montmorillonite [27,28], HNTs exhibit better dispersion properties and can help in optimizing the pore structure of membranes and are thus suitable for water treatment. Zhang et al. prepared a chemically modified hydrophilic PVDF membrane by incorporating an HNT, tannic acid, and ferric chloride for modification purposes [29]. The incorporation of the HNT provided structural reinforcement and filling effects to the membrane matrix, while the abundant Si-OH and Al-OH groups on the HNT significantly enhanced the hydrophilicity of the PVDF membrane. As an additive in the modified PVDF membrane, the HNT notably improved the membrane’s hydrophilicity, permeability and fouling resistance. The membrane demonstrated promising application prospects in the treatment of dye and antibiotic wastewater, suggesting that HNTs hold broad application potential in membrane material research. As such, employing HNTs to enhance membrane separation performance is promising when a hydrophilic modification is applied for MD membranes.

Herein, an HNT was employed for the first time to improve the antiwettability and stability of a PTFE membrane by depositing a hydrophilic mixed matrix layer to form a hydrophilic–hydrophobic dual-layer PVA-HNT/PTFE membrane. PVA was utilized as the polymer matrix due to its hydrophilicity, good film-forming properties and compatibility with HNTs [30]. The hydrophilic layer was systematically characterized to reveal the physicochemical properties before and after the incorporation of the HNT. To evaluate antifouling and antiwetting properties, the as-prepared dual-layer membrane was applied in the desalination of 3.5 wt% sodium chloride (NaCl) solution containing SDS or HA via DCMD. Moreover, the membrane with the optimal HNT loading was further applied to the treatment of landfill leachate. To date, no previous studies have reported the fabrication of a PTFE membrane modified with a PVA-HNT hydrophilic coating for MD applications. This work verifies the feasibility of the as-developed dual-layer structure, which endows the membrane with superior antiwetting and antifouling performance while maintaining operational stability.

## 2. Materials and Methods

### 2.1. Materials

PVA (Mw = 96,000 g/mol), SDS and sulfosuccinic acid (SSA) of 70 wt% were purchased from Macklin Biochemical Co., Ltd. (Shanghai, China). NaCl and HA were sourced from Tianjin Damao Chemical Reagent Factory (Tianjin, China). HNTs (typically with lengths in the range of 1–15 μm and outer diameters of approximately 50–300 nm) were acquired from XFNANO Materials Tech Co., Ltd. (Nanjing, China). All chemicals were of analytical quality and utilized as they were received. A commercially available hydrophobic PTFE membrane (pore size of 0.45 μm) was supplied by Membrane Solutions LLC (Auburn, WA, USA). For membrane antifouling evaluation, landfill leachate was provided by Suzhou Litree Ultra-Filtration Membrane Technology Co., Ltd. (Suzhou, China). Its electrical conductivity and chemical oxygen demand were 20.5 mS/cm and 1250 mg/L, respectively.

### 2.2. Synthesis of PVA-HNT/PTFE Membrane

**Figure 1:**
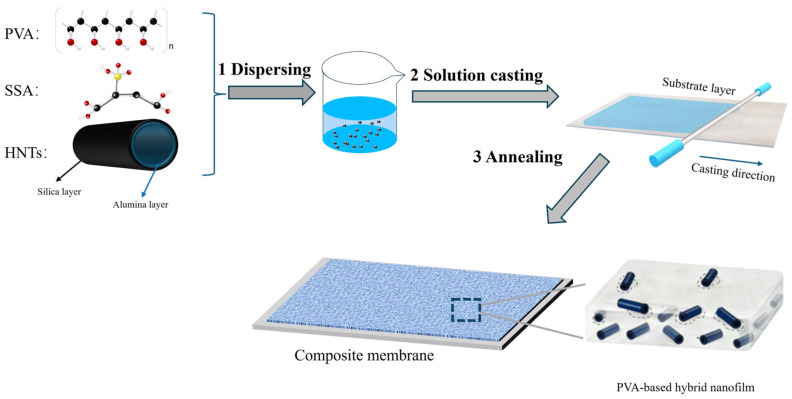
Schematic diagram of dual-layer membrane preparation.

The preparation process of the PVA-HNT/PTFE membrane is shown in Figure 1. Specifically, PVA powder (6 g) was mixed with 94 mL of deionized (DI) water under vigorous stirring at room temperature for 1 h to prepare a PVA dispersion. The mixture was then transferred to a silicone oil bath at 95 °C and continuously stirred until complete dissolution. The resulting 6 wt% PVA aqueous solution was cooled to room temperature. Subsequently, the crosslinking agent SSA (with a weight content of 20 wt% relative to PVA) was incorporated into the aqueous PVA-containing solution, and stirring was maintained for 10 min. As for HNT incorporation, 1 g of the HNT was dispersed in 99 g of DI water and sonicated in a water bath for 3 h to obtain a HNT dispersion. Predetermined amounts of the HNT dispersion were then added dropwise to the prepared aqueous solution containing PVA and SSA, followed by stirring at room temperature for 30 min and sonication for 30 min to obtain PVA solutions with different HNT concentrations (1 wt%, 5 wt%, 7 wt%, and 10 wt% with respect to PVA). The PVA-SSA-HNT aqueous mixture was then subjected to a simple solution casting method. A certain volume of the liquid mixture was cast onto the PTFE substrate at a rate of 5 mm/s using a 6 μm gap casting bar. The resulting liquid film was dried at room temperature for 30 min. The casting process was repeated two times to avoid any defects of the hydrophilic layer. Finally, the dried membrane was thermally treated in a convection oven at 95 °C for 30 min following by immersion in DI water for a certain period of time to remove unreacted crosslinkers. The dual layer membranes were named PTFE-PA for PVA-SSA on PTFE without HNT addition, PTFE-PT-1 for PVA-SSA-HNT (1 wt%) on PTFE, PTFE-PT-5 for PVA-SSA-HNT (5 wt%) on PTFE, PTFE-PT-7 for PVA-SSA-HNT (7 wt%) on PTFE, and PTFE-PT-10 for PVA-SSA-HNT (10 wt%) on PTFE. For material characterization, free-standing PVA-SSA-HNT membranes were also prepared by drying the aqueous mixture on a polystyrene Petri dish with subsequent heat treatment. The free-standing membrane samples were denoted as PA, PT-1, PT-5, PT-7, and PT-10, respectively.

### 2.3. DCMD Performance Investigation

**Figure 2:**
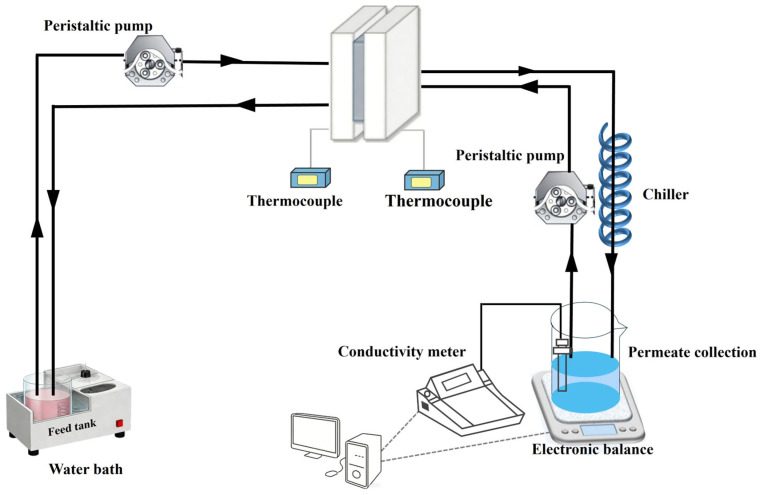
Diagram of DCMD experimental setup.

Figure 2 displays a schematic laboratory-scale diagram for the DCMD setup. The system incorporates a membrane module (membrane area of 21.5 cm^2^), equipped with thermocouples for real-time temperature monitoring. The feed and permeate streams are circulated in a counterflow manner by two peristaltic pumps. To avoid fluid leakage, high-vacuum silicone grease is applied to seal the membrane edges on both the feed and permeate sides. The flow rates on both sides are maintained at 100 ± 10 mL/min throughout all the DCMD runs. The feed inlet temperature is thermostatically controlled at 50 °C using a constant-temperature water bath, while the permeate side is kept at 10 °C by a recirculating chiller. Water vapor flux and permeate conductivity were continuously recorded using a digital balance (A&D, Model GF-6000, A&D Company, Tokyo, Japan) and a conductivity meter (Hanna HI98192, Hanna Instruments, Woonsocket, RI, USA) linked to a computer via a data acquisition system. The separation performance of the membranes was assessed using a NaCl aqueous solution (3.5 wt%) as the feed solution for a 3 h DCMD run. Antiwetting resistance was evaluated using the NaCl solution combined with SDS (0.4 mM). Furthermore, the long-term durability of the membrane was further validated via landfill leachate treatment. At least 3 independent membrane samples were used for performance testing, and the average data were calculated with standard deviation. Water flux (*J*) was calculated according to the following equation:

(1) $$J=\frac{\Delta M}{At}$$

where *J* denotes water flux, Δ*M* represents the mass change in the permeate (kg), A refers to the effective membrane area (m^2^) and *t* indicates the time interval (h) during the permeate mass change.

The salt rejection (*R*) of the dual-layer membranes was estimated using Equation (2):

(2) $$R=(1-\frac{C_{p}}{C_{f}})\times 100\%$$

where *C* is the salt concentration in either the feed (*C_f_*) or the permeate (*Cp*).

### 2.4. Characterization of Hydrophilic Layer

The micro-morphologies of the membranes, including the surface and cross-section, were observed using a field-emission scanning electron microscope (FESEM, Zeiss Sigma 300, Carl Zeiss AG, Oberkochen, Germany). The wettability of the as-prepared dual-layer membranes, in terms of hydrophilicity and hydrophobicity, was investigated by conducting water contact angle (WCA) measurements using an optical tensiometer (KRÜSS DSA30S, KRÜSS GmbH, Hamburg, Germany). The chemical structures of the HNT and the PVA-based layers were evaluated using attenuated total reflectance–Fourier transform infrared (ATR-FTIR) spectroscopy (Thermo Fisher Nicolet iS50, Thermo Fisher Scientific, Waltham, MA, USA). The effect on crystalline structure change before and after the addition of the HNT was investigated using an X-ray diffractometer (Shimadzu XRD-6100 X-ray diffractometer, Kyoto, Japan). Differential scanning calorimetry (DSC, Netzsch DSC 214, NETZSCH-Gerätebau GmbH, Selb, Germany) studies were conducted in a nitrogen environment from 10 to 250 °C with a heating rate of 10 °C/min. The LEP of the membranes was determined by pressurizing DI water onto the PVA-based layer side of PVA-HNT/PTFE membrane specimens assembled within a filtration cell. The imposed pressure was progressively elevated at an interval of 0.1 bar min^−1^ until the initial permeation droplet emerged from the permeate side. The mechanical properties of the PVA-based layer were measured (CMT 4503, Sansi Yongheng Technology Co., Ltd., Ningbo, China) to explore the influence of the HNT on the polymer matrix. The results of WCA, LEP and mechanical property measurements were evaluated with multiple replicates (*n* ≥ 3), and the results are calculated with standard deviation.

## 3. Results

### 3.1. Characterization of HNT Nanofiller

**Figure 3:**
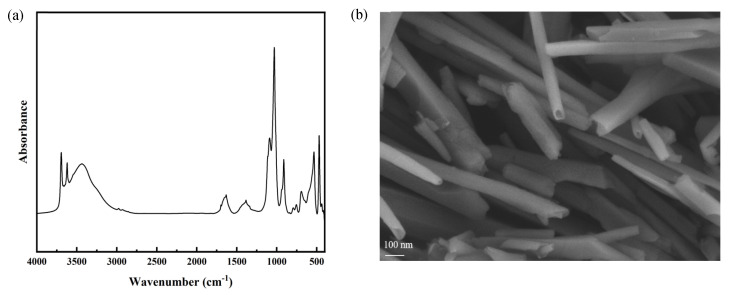
(**a**) ATR-FTIR of HNT; (**b**) SEM image of HNT.

As shown in Figure 3a, the ATR-FTIR spectrum of the HNT exhibits characteristic vibrational bands consistent with the aluminosilicate structure. In the high-frequency region, the sharp shoulder-like peaks observed at 3695 and 3622 cm^−1^ are assigned to the stretching vibrations of inner surface and inner hydroxyl groups, respectively, while the broad absorption band centered at 3450 cm^−1^ corresponds to the O-H stretching of physically adsorbed water [31]. The dominant absorption band at 1030 cm^−1^ is attributed to the in-plane Si-O-Si stretching vibrations of the tetrahedral silicate framework. Additionally, the peak at 910 cm^−1^ represents the Al-OH bending vibration of inner hydroxyls, while the low-frequency bands at 530 and 470 cm^−1^ are associated with Al-O-Si and Si-O-Si bending deformations [32]. These well-defined peaks confirm the chemistry of the HNT.

As shown in Figure 3b, the SEM image reveals the morphology of the pure HNT, exhibiting a highly uniform, straight and elongated hollow tubular structure with high aspect ratios. The nanotubes possess smooth external surfaces with no visible surface defects and well-defined open lumina, which are clearly visible at the distal ends of several tubes. No significant surface impurities or amorphous clusters are observed, suggesting the high purity and successful preservation of the cylindrical rolled-layer morphology characteristic of the 1:1 phyllosilicate structure. The 1D nanotube geometry arises from the rolling of aluminosilicate nanosheets, driven by the lattice mismatch between the gibbsite-like Al(OH)_3_ octahedral sheet (inner surface) and the silicate SiO_4_ tetrahedral sheet (outer surface).

### 3.2. Effects of HNT on PT Layer

**Figure 4:**
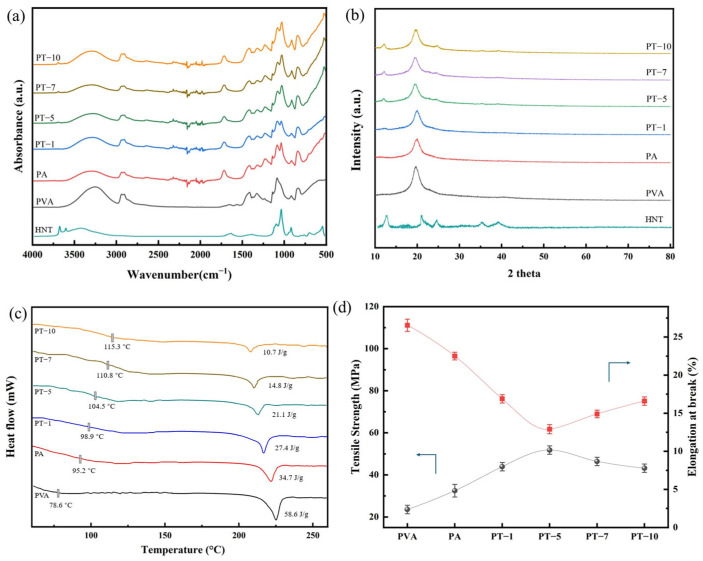
(**a**) ATR-FTIR spectrum of PVA-based films and HNT; (**b**) XRD pattens of PVA-based films and HNT; (**c**) DSC curves of PVA-based films and (**d**) tensile strength and elongation at break of films.

As shown in Figure 4a, the functional structures of PVA, PA and PT were analyzed by ATR-FTIR. For pure PVA, the characteristic absorption band of hydrogen-bonded -OH groups can be identified in the range of 3000–3600 cm^−1^ [33]. The asymmetrical and symmetrical stretching peaks of -CH groups are located at 2940 cm^−1^ and 2900 cm^−1^, respectively. The bending vibration peak of CH_2_ (derived from the PVA backbone) is observed at 1416 cm^−1^, while the C-O peak corresponding to -OH groups is found at 1085 cm^−1^. For the PA sample, characteristic peaks for C-S, S-OH, and S=O groups appear at 833 cm^−1^, 1037 cm^−1^, and 1220 cm^−1^, respectively, indicating the presence of SSA in the polymer matrix. Additionally, absorption bands corresponding to ester groups (C=O and C-O) are shown at 1710 cm^−1^ and 1272–1155 cm^−1^, respectively, suggesting the formation of covalent linkage between PVA chains via SSA bridging [34]. In the meanwhile, rocking vibrations of C-C and C-H (~510 cm^−1^) emerge after the crosslinking of PVA chains, in line with a previous study [35]. Upon the incorporation of the HNT nanofiller, the ATR-FTIR spectra of PT-1, PT-5, PT-7 and PT-10 exhibit enhanced intensities of the absorption bands of hydrogen-bonded -OH groups. Furthermore, sharp peaks associated with Al-O bending vibrations can be found at 520 cm^−1^. These changes in ATR-FTIR spectra confirm the incorporation of the HNT into the polymer matrix.

As shown in Figure 4b, the phase structures of the prepared membranes were investigated by XRD. All PVA-based samples displayed characteristic (101) diffraction peaks at 19.7°, corresponding to the orthorhombic (101) lattice plane that confirmed the semi-crystalline nature of PVA bearing both ordered crystalline domains and disordered amorphous regions. After crosslinking with SSA, the full width at half maxima (FWHM) of the PA membrane increased from 1.42 to 2.2 (2θ), indicating a reduction in the short-range regular packing of polymer chains. This was caused by the crosslinking reactions between SSA and PVA that disrupted regular chain packing. After the incorporation of the HNT, diffraction peaks located at 12.3° and 24.3° were present, which are associated with the characteristic crystal planes (001) and (002) of the HNT. The presence of these diffraction peaks confirmed the introduction of the HNT into the polymer matrix. Furthermore, the FWHM progressively increased with HNT loading, i.e., 2.4 (2θ) for PT-1, 2.6 (2θ) for PT-5, 2.8 (2θ) for PT-7 and 2.9 (2θ) for PT-10, indicating the gradual disruption of PVA chain packing due to the presence of the HNT in the polymer matrix. One of the main reasons is the physical obstruction posed by the HNT, restricting the growth of PVA crystallites. In addition, strong interfacial interactions between the HNT and PVA also play a significant role in restraining the formation of crystalline domains.

Figure 4c displays DSC thermograms that reflect the thermal properties of the membranes. Pristine PVA exhibited a glass transition temperature (T_g_) of 78.6 °C, which increased to 95.2 °C after crosslinking with SSA (PA), indicating restricted segmental mobility due to the formation of a chemically crosslinked network. With the introduction of the HNT, T_g_ further increased progressively from 98.9 °C for PT-1 to 115.3 °C for PT-10, suggesting that the interfacial interactions between PVA chains and HNT surfaces further constrained polymer chain dynamics. The increase in T_g_ signifies the transition of the polymer towards a more rigid, glassy state that requires more thermal energy to initiate segmental motion. On the other hand, melting enthalpy (ΔH_m_) decreased markedly from 58.6 J/g for PVA to 34.7 J/g for PA and further down to 10.7 J/g for PT-10, indicating a substantial reduction in the crystallization behavior of PVA [36]. These results indicated that both crosslinking and HNT incorporation disrupted the regular packing of PVA chains, suppressing crystal growth and promoting the formation of a more amorphous structure. In addition, the melting endotherm becomes progressively broader and less intense with increasing HNT loading, implying reduced crystal size and structural heterogeneity. For PT membranes, the combined effects of crosslinking and HNT incorporation gave rise to a more rigid yet amorphous polymer matrix, which might be beneficial for providing more permeable areas while maintaining selectivity.

The mechanical properties of the membranes are shown in Figure 4d. The PVA membrane exhibited a relatively low tensile strength of 23.5 MPa but a high elongation at break of 26.6%. Upon crosslinking with SSA, tensile strength increased to 32.8 MPa, whereas elongation at break decreased to 22.4%. This indicated that crosslinking introduced a more rigid network structure, enhancing mechanical strength at the expense of flexibility due to restricted chain mobility. With the incorporation of the HNT, tensile strength increased significantly, reaching a maximum at 51.8 MPa for PT-5, followed by a slight decline at higher loadings. In contrast, elongation at break decreased with the HNT content, dropping to a minimum of 12.9% for PT-5 and then showing a slight increase at higher loadings. At HNT loadings ≤5 wt%, well-dispersed HNTs acted as reinforcing nanofillers, promoting stress transfer between the polymer matrix and the rigid nanotubes, thereby significantly enhancing tensile strength. Simultaneously, strong interfacial interactions restricted chain mobility, leading to reduced elongation at break. At higher HNT loadings (>5 wt%), the slight decrease in tensile strength and partial recovery in elongation suggested the onset of nanotube aggregation. Such aggregation could create microstructural heterogeneities and reduce effective stress transfer efficiency while locally loosening the polymer network.

### 3.3. Microstructural Characteristics of PVA-HNT/PTFE Dual-Layer Membranes

**Figure 5:**
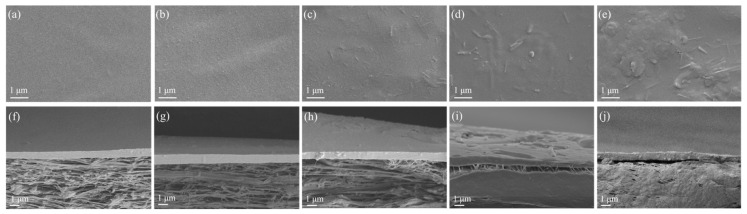
SEM images of dual-layer membranes: (**a**) surface view of PA, (**b**) surface view of PTFE-PT-1, (**c**) surface view of PTFE-PT-5, (**d**) surface view of PTFE-PT-7, (**e**) surface view of PTFE-PT-10, (**f**) cross-sectional view of PA, (**g**) cross-sectional view of PTFE-PT-1, (**h**) cross-sectional view of PTFE-PT-5, (**i**) cross-sectional view of PTFE-PT-7, (**j**) cross-sectional view of PTFE-PT-10.

SEM was employed to observe the micro-scale morphologies of the dual-layer membranes before and after the addition of the HNT nanofiller. The PTFE-PA membrane (Figure 5a) exhibited a smooth and dense surface, which could be attributed to the dense polymer nature of PVA. At 1 wt% loading of the HNT (Figure 5b), the membrane surface remained relatively smooth and uniform, implying that HNT nanofillers were well dispersed within the PVA matrix without significantly disturbing the polymer network. As the HNT content increased to 5 wt% (Figure 5c), rod-like features that were attributed to the HNT became discernible, indicating partial exposure or near-surface distribution. At 7 wt% loading, small aggregates and surface irregularities emerged, suggesting the onset of HNT agglomeration. Further increasing the loading of the HNT to 10 wt% (Figure 5e) caused more pronounced aggregation and surface roughening, accompanied by the formation of clusters and protrusions, implying reduced compatibility and filler dispersion with a possible HNT-rich phase and polymer-rich phase.

The cross-sectional images (Figure 5f–j) further confirmed the above-mentioned observations. The PTFE-PA membrane (Figure 5f) showed a thin, continuous, and defect-free selective layer adhered to the PTFE substrate. For the PTFE-PT-X membranes, the selective layer remained continuous, but its interface became more irregular with increasing HNT loading, indicating the influence of nanofiller incorporation on membrane structure. The thicknesses of the hydrophilic layers were in the range of 1.3–1.6 μm. The SEM results revealed that low to moderate HNT incorporation (1 wt% to 5 wt%) promoted relatively uniform dispersion without compromising membrane integrity, whereas excessive loading (7 wt% to 10 wt%) induced aggregation and increased surface roughness.

### 3.4. Surface Properties of PVA-HNT/PTFE Dual-Layer Membranes

**Figure 6:**
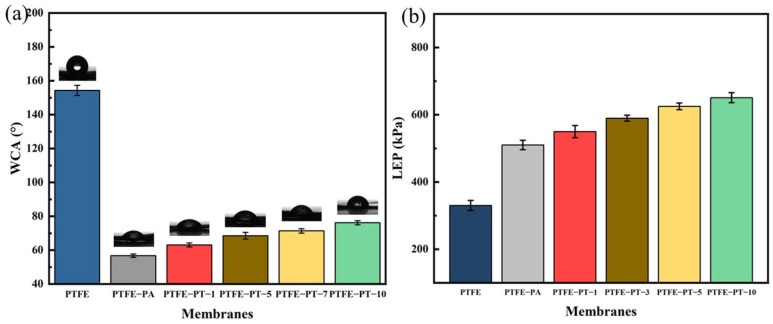
(**a**) WCAs of membranes and (**b**) LEP of membranes.

The hydrophilicity and hydrophobicity of the membranes were examined by WCA measurements with the PTFE membrane for comparison. As shown in Figure 6a, PTFE exhibited a high WCA of 154.6°, which was attributed to the low surface energy of fluorinated groups. The WCA of PTFE-PA dramatically decreased to 56.8°, indicating a transition to a hydrophilic surface. This substantial reduction in WCA values could be ascribed to the presence of hydroxyl groups in PVA and the hydrophilic sulfonic acid functionalities introduced by SSA. With the incorporation of the HNT into the PVA matrix (PTFE-PT-X membranes), the WCA gradually increased from 63.1° to 76.2° as HNT loading rose from 1 to 10 wt%, indicating a transition toward greater hydrophobicity. This phenomenon could be attributed to multiple factors that collectively affect surface hydrophilicity, including the crosslinking of PVA, surface morphology and the dispersion state of the HNT. Although the HNT is inherently hydrophilic, its surface chemistry may vary depending on its source and subsequent processing conditions, resulting in possibly lower hydrophilicity compared to PVA. Additionally, the aggregation of HNT particles led to the shielding of effective HNT surfaces, reducing the availability of surface hydrophilic groups. As evidenced by the SEM images, the introduction of the HNT induced a rougher membrane surface. According to the Wenzel model, such surface roughness could further amplify the apparent hydrophobicity if air pockets are partially entrapped between the membrane surface and the water droplet [37]. Therefore, these aforementioned factors might synergistically contribute to the variations in surface WCA observed for the PTFE-PT-X membranes.

The impact of the HNT on the LEP of the membranes is presented in Figure 6b. The pristine PTFE membrane exhibited a relatively low LEP of 330 kPa, which was mainly ascribed to its inherently open and highly porous structure. Once the applied pressure exceeds the LEP value of the PTFE substrate, liquid can penetrate into the internal pores and induce membrane wetting. After the deposition of the PA layer, the LEP increased remarkably to 510 kPa. Such a noticeable improvement was attributed to the attachment of a dense and nonporous PVA-based layer that effectively blocked water transport pathways and prevented direct liquid intrusion into PTFE pores. Thus, the introduction of the PVA-based top layer substantially raised the critical hydraulic pressure for liquid penetration. After incorporating the HNT, the LEP showed a progressive increase from 550 kPa (PTFE-PT-1) to a maximum of 650 kPa (PTFE-PT-10). The enhanced LEP originated from interfacial interactions between the HNT and PVA, which likely reinforced the polymer network and improved mechanical stability, as evidenced in the mechanical strength results. At an HNT loading of 10 wt%, no reduction in LEP was observed, indicating that no severe phase separation occurred, even though HNT agglomeration was evident in the SEM images.

### 3.5. DCMD of PVA-HNT/PTFE Dual-Layer Membranes

**Figure 7:**
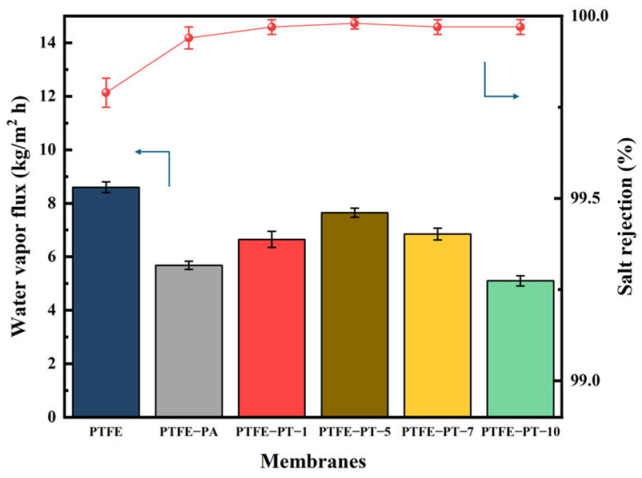
DCMD performance of PTFE and dual-layer membranes.

The water vapor flux and salt rejection of the membranes during DCMD using a 3.5 wt% NaCl solution as the feed are presented in Figure 7. The bare PTFE membrane possessed the highest water vapor flux of 8.6 kg/m^2^ h, which can be attributed to its intrinsic hydrophobicity, large pore size, and minimal vapor mass transfer resistance. After coating with the PA layer, the water vapor flux of PTFE-PA dropped significantly to 5.6 kg/m^2^ h. This decline was primarily due to the introduction of a dense hydrophilic selective layer atop, which added mass transfer resistance by reducing effective vapor transport pathways. With the incorporation of the HNT, the flux exhibited a non-monotonic up-and-down-like evolution. At a low loading of 1 wt% HNT (PTFE-PT-1), the flux increased to 6.6 kg/m^2^ h. The increase in water vapor flux was mainly attributed to the decrease in the crystallization of PVA, which provided more amorphous regions, i.e., increased permeable membrane area. In addition, the presence of the HNT might also contribute to the enhancement in water vapor flux due to the tubular lumen structure, providing additional straight nanochannels rather than tortuous diffusion compared to the pure polymer phase. This could enhance water transport efficiency through the hydrophilic layer. The flux reached a maximum at 7.6 kg/m^2^ h for PTFE-PT-5, indicating an optimal filler loading where the formation of additional transport pathways and improved microstructure outweighed the resistance introduced by the coating layer. However, further increasing HNT loading (PTFE-PT-7 and PTFE-PT-10) caused a decline in flux (6.8 and 5.1 kg/m^2^ h, respectively). This could be due to the formation of HNT aggregation, reducing the effective polymer–nanofiller interface with the possible blockage of transport channels. In addition, possible phase separation might also reduce the water transport rate, resulting in the declined throughput of the membrane.

In contrast, salt rejection remains consistently high for all modified membranes (>99.9%), compared to slightly lower rejection for pristine PTFE (99.2%). The enhanced rejection of coated membranes can be attributed to the dense PVA-based selective layer, which effectively prevented liquid penetration and salt passage. Notably, the incorporation of the HNT slightly improved rejection further, reaching nearly 99.95% for PTFE-PT-5, indicating improved structural integrity and antiwetting performance. Even at higher HNT loadings, rejection remains stable, suggesting that no significant defects were introduced despite the presence of filler aggregations. Among the dual-layer membranes, the PTFE-PT-5 membrane demonstrates the best performance, achieving a favorable balance between high water vapor flux and near-complete salt rejection. Furthermore, a comprehensive comparison with recently reported dual-layer MD membranes was conducted (Table S1) to further evaluate the performance of the PTFE-PT-5 membrane [38,39,40,41,42,43,44,45,46,47]. The PTFE-PT-5 membrane achieves a promising balance between water vapor flux and salt rejection, exhibiting separation performance comparable to that of state-of-the-art MD membranes.

### 3.6. Antiwetting Properties of PVA-HNT/PTFE Dual-Layer Membranes

**Figure 8:**
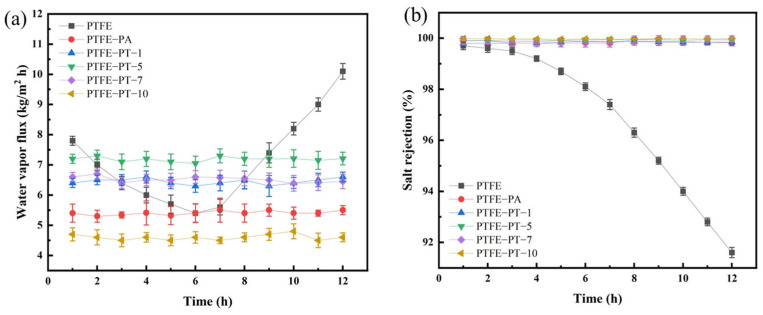
Performance of membranes with respect to SDS-containing solutions: (**a**) water vapor flux and (**b**) salt rejection.

As illustrated in Figure 8, antiwetting ability was evaluated using a NaCl solution containing 0.4 mM SDS, which imposed severe wetting conditions owing to the reduced surface tension of the solution. For the pristine PTFE membrane, its flux decreased from 7.8 to 5.4 kg/m^2^·h within the initial 6 h of operation, followed by a successive increase to 10.1 kg/m^2^·h. These flux variations were attributed to the sequential occurrence of the surface wetting, partial wetting, and full wetting of the hydrophobic pores in the PTFE membrane. In contrast, all modified membranes (PTFE-PA and PTFE-PT-X series) maintained relatively stable fluxes (5.4 kg/m^2^·h for PTFE-PA, 6.4 kg/m^2^·h for PTFE-PT-1, 7.2 kg/m^2^·h for PTFE-PT-5, 6.6 kg/m^2^·h for PTFE-PT-7 and 4.6 kg/m^2^·h for PTFE-PT-10) throughout the entire DCMD process, demonstrating the effective suppression of membrane wetting. In the meanwhile, the salt rejection results further confirmed the enhanced wetting resistance of the dual-layer membranes. Over the course of the experiment, the pristine PTFE membrane exhibited a significant decline in salt rejection, dropping from 99.5% to 91.5%, which clearly evidenced severe wetting induced by SDS adsorption. On the other hand, the PTFE-PA membrane and all PTFE-PT-X membranes sustained consistently high salt rejection rates (ranging from 99.80% to 99.95%) throughout the 12 h test period, indicating excellent antiwetting stability.

The enhanced antiwetting performance of the PVA-based dual-layer membranes can be ascribed to the synergistic effects of the hydrophilic PVA-based layer and the incorporation of the HNT. Specifically, the PVA-based layer formed a hydration barrier on the membrane surface due to its hydrophilic nature, which not only mitigated the adsorption of SDS molecules but also inhibited the penetration of liquid into the hydrophobic PTFE substrate. Meanwhile, the PVA-based coating effectively increased the LEP of the membrane as evidenced in Section 3.4, ensuring enhanced wetting resistance. The incorporation of the HNT not only facilitated water transport but also improved the structural stability of the hydrophilic layer. Consequently, these results demonstrated that the pristine PTFE membrane was highly susceptible to conventional surfactant-induced wetting, whereas the dual-layer membranes, particularly that with an optimal HNT loading (5 wt%), exhibited superior wetting resistance while maintaining high permeability and near-complete salt rejection. This excellent antiwetting performance demonstrated the critical role of HNT incorporation in achieving balanced performance between flux enhancement and antiwetting stability for DCMD applications. Furthermore, the antifouling performance of the PTFE-PT-5 and pristine PTFE membranes was evaluated using a saline solution containing 10 mg/L humic acid (HA) and 3.5 wt% NaCl. The results presented in Figure S1 verified that the PTFE-PT-5 membrane possessed outstanding antifouling capability, which can be attributed to the presence of the PVA-based hydrophilic layer.

### 3.7. Long-Term Stability Comparison

**Figure 9:**
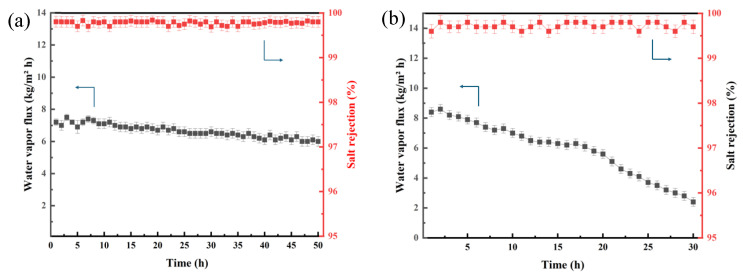
Long-term performance of (**a**) PTFE-PT-5 and (**b**) PTFE.

**Figure 10:**
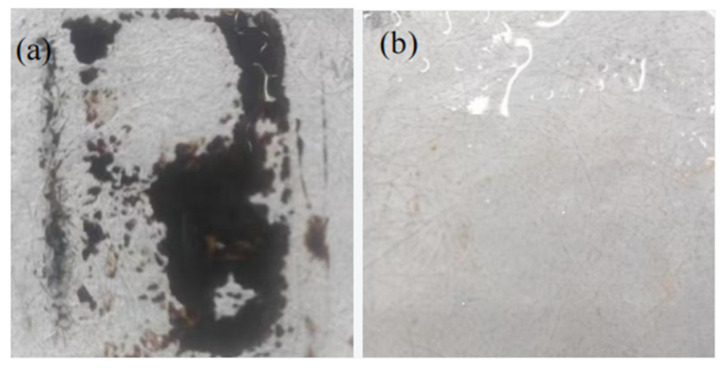
Surface observation of membranes after long-term test: (**a**) PTFE and (**b**) PTFE-PS-5.

Since the PTFE-PT-5 membrane exhibited the best overall performance, DCMD performance was further evaluated using real landfill leachate as the feed to assess the long-term stability of the membranes. As shown in Figure 9a, the PTFE-PT-5 membrane exhibits highly stable desalination performance over the 50 h of operation. The water vapor flux remained nearly constant, with a decline of 15.3% (7.2 to 6.1 kg/m^2^·h), which can be attributed to mild fouling or partial surface coverage by organic/inorganic constituents present in the leachate. Importantly, salt rejection was consistently maintained at around 99.95% throughout the entire testing period, indicating the absence of pore wetting and excellent integrity of the selective layer. In contrast, the pristine PTFE membrane (Figure 10b) showed a pronounced deterioration in DCMD performance. The water vapor flux decreased continuously from 8.5 to 2.5 kg/m^2^·h over 30 h operation time, i.e., a 70.5% decline, suggesting severe fouling and progressive pore blockage. Meanwhile, although salt rejection remained relatively high (>99%) in the initial stage, a slight downward fluctuation was observed at later stages, implying the onset of partial wetting or compromised selectivity. The simultaneous decline in flux and instability in rejection confirmed that the pristine PTFE membrane was highly susceptible to the complex foulants and surfactants present in landfill leachate.

After the DCMD test, the surface of the membranes was observed visually, as shown in Figure 10. It can be seen that the surface of PTFE exhibited extensive and non-uniform accumulation of dark, carbonaceous foulants, indicating the formation of a dense fouling layer. The localized nature of the deposits suggested the presence of hydrophobic–hydrophobic interactions between PTFE and organic constituents in the landfill leachate, leading to membrane fouling and pore blockage. In contrast, the PTFE-PT-5 membrane surface remained remarkably clean, maintaining a visual appearance nearly identical to its pre-test state. The absence of significant foulant deposition demonstrated a substantial enhancement in the fouling resistance of the PTFE-PT-5 membrane. The thin PVA-based top layer effectively acted as a protective barrier, preventing the direct infiltration of leachate contaminants into the underlying PTFE microporous structure. While PTFE was inherently hydrophobic and prone to organic adsorption, the PT layer introduced hydrophilic groups into the membrane interface. This facilitated the formation of a hydration layer via hydrogen bonding with water molecules, thereby forming an energy barrier that effectively sterically hindered the attachment of hydrophobic foulants while allowing for selective water transport. Overall, apart from the superior antifouling and antiwetting performance, the simplicity of the solution-casting process, combined with the low cost and natural abundance of HNTs, suggests promising potential for the scalable and economically feasible production of this dual-layer membrane.

## 4. Conclusions

In summary, hydrophilic–hydrophobic dual-layer membranes based on PVA-HNT/PTFE were developed for the DCMD process by coating a PVA-based layer containing HNT nanofiller onto the surface of a microporous PTFE membrane. The PVA matrix was modulated to exhibit reduced melting enthalpy, elevated T_g_ and increased FWHM, which collectively indicated diminished crystallization and chain rigidification by the incorporation of the HNT. The as-prepared PT layer displayed HNT-regulated water transport behavior, wherein the PTFE-PT-5 membrane achieved optimal performance, with a water vapor flux enhancement of 35.7% compared to the HNT-free dual-layer membrane (PTFE-PA). Owing to the presence of the hydrophilic coating layer, the effect of wetting agents was efficiently avoided during the DCMD process, thereby endowing the membrane with prominent antiwettability against low-surface-tension saline solutions. More importantly, DCMD tests for landfill leachate treatment demonstrated that the PTFE-PT-5 membrane possessed superior antifouling capabilities with respect to highly contaminated wastewater, achieving stable flux and near-complete salt rejection during prolonged operation. In contrast, the pristine PTFE membrane underwent severe fouling and significant performance deterioration under identical operating conditions. This study underscores the role of hydrophilic layer and nanofiller incorporation in enhancing membrane durability under practical and challenging feedwater conditions.

## Data Availability

The original contributions presented in the study are included in the article. Further inquiries can be directed to the corresponding author.

## Supplementary Materials

The following supporting information can be downloaded at https://www.mdpi.com/article/10.3390/membranes16060201/s1, Figure S1. Performance of PTFE and PTFE-PT-5 towards HA containing solution (NaCl concentration: 3.5 wt%; HA concentration: 10 mg/L). Table S1. Comparison of the PTFE-PT-5 with reported dual-layer MD membranes.

## References

[1] Julian H., Nurgirisia N., Qiu G., Ting Y.-P., Wenten I.G. (2022). Membrane distillation for wastewater treatment: Current trends, challenges and prospects of dense membrane distillation. J. Water Process Eng..

[2] Razaqpur A.G., Wang Y., Liao X., Liao Y., Wang R. (2021). Progress of photothermal membrane distillation for decentralized desalination: A review. Water Res..

[3] Zhou J., Li Y., Wu Q., Liu Z., Zhang M., Hu H., Wang X., Lu X., Wu C. (2025). Design of a superhydrophobic network layer for enhancing the stability and wetting resistance of membrane distillation. Sep. Purif. Technol..

[4] Lou M., Meng L., Li J., Li F. (2025). Review of designing anti-surfactant wetting Janus membranes for membrane distillation: Mechanisms, methods and challenges. Desalination.

[5] Alkhatib A., Jiang H., Du F., Stolte S., Alkhamri K.A., Ayari M.A., Wang Y., Hawari A.H. (2025). Enhancement of membrane distillation in seawater desalination process using inhomogeneous electric field. Desalination.

[6] Nurjanah T.-T., Chang S.-J., You C.-Y., Huang W.-Y., Sean (2024). Reverse osmosis integrated with renewable energy as sustainable technology: A review. Desalination.

[7] Xiao H., Feng Y., Goundry W.R.F., Karlsson S. (2024). Organic Solvent Nanofiltration in Pharmaceutical Applications. Org. Process Res. Dev..

[8] Al-Jariry N., Morosini E., Lipnizki F., Carraretto I.M. (2026). Analysis of high flux membranes for desalination in waste-heat driven vacuum membrane distillation plants: Experimental validation and techno-economic analysis. Desalination.

[9] Moreira V.R., Raad J.V., Lazarini J.X., Santos L.V.S., Amaral M.C.S. (2023). Recent progress in membrane distillation configurations powered by renewable energy sources and waste heat. J. Water Process Eng..

[10] Christie K.S.S., Horseman T., Lin S. (2020). Energy efficiency of membrane distillation: Simplified analysis, heat recovery, and the use of waste-heat. Environ. Int..

[11] Zhang T., Laborie S., Cabassud C. (2022). Optical method for in operando study of membrane distillation wetting: Development for desalination and potential for exploring wetting dynamics. Desalination.

[12] Li H., Yu Z., Lin Y., Zhang W., Lai C., Xia L., Meng F., Zhao S. (2025). Janus membrane with simultaneously robust scaling, wetting and fouling resistance for hypersaline organic wastewater treatment via membrane distillation. J. Membr. Sci..

[13] Moriones A., Cano-Herranz L., Luque-Alled J.M., Téllez C., Gorgojo P. (2025). Metal-organic frameworks/graphene oxide nanohybrids to control pore wetting in membrane distillation. Desalination.

[14] Chang H., Huang Y., Gu S., Cheng X., Qu F. (2026). Role of multivalent iron in enhancing sodium percarbonate oxidation for alleviating membrane distillation fouling caused by shale gas produced water. Water Res..

[15] Lin P.-J., Yang M.-C., Li Y.-L., Chen J.-H. (2015). Prevention of surfactant wetting with agarose hydrogel layer for direct contact membrane distillation used in dyeing wastewater treatment. J. Membr. Sci..

[16] Wang Z., Chen Y., Sun X., Duddu R., Lin S. (2018). Mechanism of pore wetting in membrane distillation with alcohol vs. surfactant. J. Membr. Sci..

[17] Jia H., Ren J., Kong Y., Ji Z., Guo S., Li J. (2024). Recent Advances in Dopamine-Based Membrane Surface Modification and Its Membrane Distillation Applications. Membranes.

[18] Sun N., Qu Y., Qian A., Li R., Zhao H., Cheng F., Li J. (2023). Improving permeability and fouling resistance of GO hydrophilic/hydrophobic Janus membrane by polyether amine crosslinking for membrane distillation of dye wastewater. J. Environ. Chem. Eng..

[19] Talebi M., Saljoughi E., Karkhanechi H., Mousavi S.M. (2026). Pore-filling method for hydrophilic-hydrophobic membranes: Enhancing performance in membrane distillation. Desalination.

[20] Li C., Li X., Du X., Tong T., Cath T.Y., Lee J. (2019). Antiwetting and Antifouling Janus Membrane for Desalination of Saline Oily Wastewater by Membrane Distillation. ACS Appl. Mater. Interfaces.

[21] Zhao L., Wu C., Lu X., Ng D., Truong Y.B., Xie Z. (2018). Activated carbon enhanced hydrophobic/hydrophilic dual-layer nanofiber composite membranes for high-performance direct contact membrane distillation. Desalination.

[22] Zhang J., Yang X., Zhang N., Tao Z., Han L., Wei B., Qi R., Fu Y., Wang Z. (2025). Polyamide membrane based on hydrophilic intermediate layer for simultaneous wetting and fouling resistance in membrane distillation. J. Membr. Sci..

[23] Li Z., Guo Z., Xie J., Zhao G., Liu Y. (2024). MOF-based photothermal Janus membrane with robust anti-fouling performance for enhanced membrane distillation. Desalination.

[24] Han D., Shuang S., Ye J., Ye H., Yang X., Liu Y. (2025). Polydopamine-Modified Halloysite Nanotube Blended Sulfonated Poly(ether Ether Ketone) Cross-Linked Composite Membrane for Fuel Cells. Energy Fuels.

[25] Cheng B., Wang Y., Wu X., Fang M., Min X., Huang Z., Liu Y., Mi R. (2022). Preparation and characterization of novel thin film composite forward osmosis membrane with halloysite nanotube interlayer. Polymer.

[26] Nigiz F.U., Karakoca B. (2023). Halloysite Nanotube doped poly lactic acid membrane preparation and seawater desalination. Appl. Clay Sci..

[27] Miao E.-D., Ye M.-Q., Guo C.-L., Liang L., Liu Q., Rao Z.-H. (2019). Enhanced solar steam generation using carbon nanotube membrane distillation device with heat localization. Appl. Therm. Eng..

[28] Wae AbdulKadir W.A.F., Ahmad A.L., Ooi B.S. (2022). Hydrophobic Montmorillonite/PVDF Membrane: Experimental Investigation of Membrane Synthesis toward Wetting Characterization and Performance via DCMD. Arab. J. Sci. Eng..

[29] Zhang R., Liu Y., Li Y., Han Q., Zhang T., Zeng K., Zhao C. (2021). Preparation of polyvinylidene fluoride modified membrane by tannin and halloysite nanotubes for dyes and antibiotics removal. J. Mater. Sci..

[30] Choi S., Chaudhari S., Shin H., Cho K., Lee D., Shon M., Nam S., Park Y. (2022). Polydopamine-modified halloysite nanotube-incorporated polyvinyl alcohol membrane for pervaporation of water-isopropanol mixture. J. Ind. Eng. Chem..

[31] Viretto A., Jasinski E., Lafon-Pham D., Otazaghine B., Sonnier R., Taguet A. (2024). A new method based on TGA/FTIR coupling to quantify the different thermal degradation steps of EVA/HNT composites prepared by different processing. J. Anal. Appl. Pyrolysis.

[32] Şomoghi R., Mihai S., Teodorescu G.-M., Vuluga Z., Gabor A.R., Nicolae C.-A., Trică B., Vătău D.M.S., Oancea F., Stănciulescu C.M. (2024). Influence of HNT-ZnO Nanofillers on the Performance of Epoxy Resin Composites for Marine Applications. Coatings.

[33] Sansone V., Macedonio F., Poerio T., Vitola G., Bruno L., Piscioneri A., Frappa M., Greco S., Romeo T., Rizzo C. (2026). Biofunctionalized and bioluminescent PVA membrane for ATP detection. J. Membr. Sci..

[34] Gomaa M.M., Hugenschmidt C., Dickmann M., Abdel-Hady E.E., Mohamed H.F.M., Abdel-Hamed M.O. (2018). Crosslinked PVA/SSA proton exchange membranes: Correlation between physiochemical properties and free volume determined by positron annihilation spectroscopy. Phys. Chem. Chem. Phys..

[35] Hendy M.A., Kashar T.I., Allam E.M., Gado M.A., Yahia N.S., Cheira M.F. (2024). Thorium ions elimination from its solution utilizing the assembled sulfosuccinic acid/polyvinyl alcohol/polyamide. Mater. Today Commun..

[36] Wang B., Chen Z., Zhang J., Cao J., Wang S., Tian Q., Gao M., Xu Q. (2014). Fabrication of PVA/graphene oxide/TiO_2_ composite nanofibers through electrospinning and interface sol–gel reaction: Effect of graphene oxide on PVA nanofibers and growth of TiO_2_. Colloids Surf. A Physicochem. Eng. Asp..

[37] Han T.-Y., Shr J.-F., Wu C.-F., Hsieh C.-T. (2007). A modified Wenzel model for hydrophobic behavior of nanostructured surfaces. Thin Solid Films.

[38] Byun S., Seo Y.J., Park C.H., Jeong S. (2026). Dual resistance Janus PDA/PVDF membrane for removal and concentration of short- and long-chain perfluoroalkyl substances via membrane distillation. Desalination.

[39] Byun S., Wong P.W., Kharraz J.A., Nam S.Y., An A.K., Jeong S. (2024). Dual resistance Janus PDA/patterned PVDF membrane for membrane distillation with early wetting detection using electrochemical impedance spectroscopy. Desalination.

[40] Huang Z., Yang G., Zhang J., Gray S., Xie Z. (2021). Dual-layer membranes with a thin film hydrophilic MOF/PVA nanocomposite for enhanced antiwetting property in membrane distillation. Desalination.

[41] Chen Y., Ju J., Zhang Y., Zhou Y., Wang Y., Kang W. (2024). Dual-structured PTFE/PI-PI/PANI composite membranes for photothermal membrane distillation with excellent photothermal conversion and open pathways for water vapor transport. Desalination.

[42] Yang G., Ng D., Huang Z., Zhang J., Gray S., Xie Z. (2023). Janus hollow fibre membranes with intrusion anchored structure for robust desalination and leachate treatment in direct contact membrane distillation. Desalination.

[43] Jia W., Kharraz J.A., Sun J., An A.K. (2021). Hierarchical Janus membrane via a sequential electrospray coating method with wetting and fouling resistance for membrane distillation. Desalination.

[44] Jia Y., Guan K., Mai Z., Fang S., Li Z., Zhang P., Zou D., Jiang X., He G., Matsuyama H. (2023). Thin continuous membrane coating with high surface energy for comprehensive antifouling seawater distillation. Water Res..

[45] Niknejad A.S., Kargari A., Namdari M., Pishnamazi M., Barani M., Ranjbari E., Sallakhniknezhad R., Bazgir S., Rasouli M., McAvoy D. (2023). A scalable dual-layer PAN/SAN nanofibrous membrane for treatment of saline oily water using membrane distillation. Desalination.

[46] Lou M., Huang S., Zhu X., Chen J., Fang X., Li F. (2024). Dual-polymers inserted graphene oxide membranes with enhanced anti-wetting and anti-scaling performance for membrane distillation. J. Membr. Sci..

[47] Tang M., Zheng L., Hou D., Jia X., Wang J. (2022). Microstructure design and construction of anti-wetting and anti-fouling multifunctional Janus membrane for robust membrane distillation. Chem. Eng. J..

