# Supplementary material for: Dual-Layer PVA-HNT/PTFE Membranes for Boosted Antiwettability and Stability in Membrane Distillation
Source: Membranes (Basel). 2026 Jun 9;16(6):201. doi: 10.3390/membranes16060201 (PMC13302807; doi:10.3390/membranes16060201)
Supplement: Supplementary file 1 [file membranes-16-00201-s001.zip › membranes-4296928-supplementary.pdf]

# Supplementary Material

# Dual-layer PVA-HNT/PTFE membranes for boosted antiwettability and stability in membrane distillation

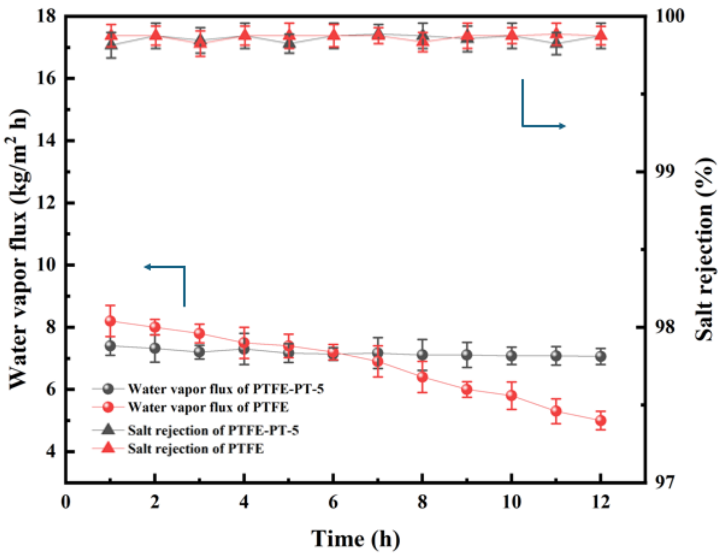


**Figure S1.** Performance of PTFE and PTFE-PT-5 towards HA containing solution (NaCl concentration: 3.5 wt%; HA concentration: 10 mg/L).

**Table S1** Comparison of the PTFE-PT-5 with reported dual-layer MD membranes.

| Hydrophilic layer | Hydrophobic layer | Configuration | Feed/permeate temperature (°C) | Water flux (kg/m$^2$ h) | Salt rejection (%) | Ref |
|---|---|---|---|---|---|---|
| Polydopamine | PVDF | DCMD | 60/20 | 26 | 99.5 | [1] |
| Polydopamine | patterned PVDF | DCMD | 60/20 | 12.50 -11.42 | 99.5 | [2] |
| PVA/AlFu MOF | PTFE | DCMD | 50/10 | 25.3 | 99.9 | [3] |
| PANI/PI | PTFE/PI | DCMD | 20/20 | 1.44 | 99.99 | [4] |
| GO/PVA/SSA | PP | DCMD | 50/10 | 4.6 | 99.5 | [5] |
| PAN nanofilaments | PVDF | DCMD | 60/20 | 22.8 | 99.99 | [6] |
| SA/PEI | PTFE | DCMD | 60/20 | 45 | 99.9 | [7] |
| PAN | SAN | DCMD | 60/25 | 34.89 | 99.9 | [8] |
| PEI/PAA/GO | PVDF | DCMD | 60/20 | 14.6 | - | [9] |
| PVA/$Al_2O_3$-APTES | PVDF/$Al_2O_3$-HTDMS-FAS | DCMD | 53/20 | 9.9 | ~100 | [10] |
| PVA-SSA-HNT | PTFE | DCMD | 50/10 | 7.6 | 99.95% | This work |

## References

[1] S. Byun, Y.J. Seo, C.H. Park, S. Jeong, Dual resistance Janus PDA/PVDF membrane for removal and concentration of short- and long-chain perfluoroalkyl substances via membrane distillation, Desalination 629 (2026).
[2] S. Byun, P.W. Wong, J.A. Kharraz, S.Y. Nam, A.K. An, S. Jeong, Dual resistance Janus PDA/patterned PVDF membrane for membrane distillation with early wetting detection using electrochemical impedance spectroscopy, Desalination 580 (2024).
[3] Z. Huang, G. Yang, J. Zhang, S. Gray, Z. Xie, Dual-layer membranes with a thin film hydrophilic MOF/PVA nanocomposite for enhanced antiwetting property in membrane distillation, Desalination 518 (2021).
[4] Y. Chen, J. Ju, Y. Zhang, Y. Zhou, Y. Wang, W. Kang, Dual-structured PTFE/PI-PI/PANI composite membranes for photothermal membrane distillation with excellent photothermal conversion and open pathways for water vapor transport, Desalination 575 (2024).
[5] G. Yang, D. Ng, Z. Huang, J. Zhang, S. Gray, Z. Xie, Janus hollow fibre membranes with intrusion anchored structure for robust desalination and leachate treatment in direct contact membrane distillation, Desalination 551 (2023).
[6] W. Jia, J.A. Kharraz, J. Sun, A.K. An, Hierarchical Janus membrane via a sequential electrospray coating method with wetting and fouling resistance for membrane distillation, Desalination 520 (2021).
[7] Y. Jia, K. Guan, Z. Mai, S. Fang, Z. Li, P. Zhang, D. Zou, X. Jiang, G. He, H. Matsuyama, Thin continuous membrane coating with high surface energy for comprehensive antifouling seawater distillation, Water Res. 244 (2023).
[8] A.S. Niknejad, A. Kargari, M. Namdari, M. Pishnamazi, M. Barani, E. Ranjbari, R. Sallakhniknezhad, S. Bazgir, M. Rasouli, D. McAvoy, A scalable dual-layer PAN/SAN nanofibrous membrane for treatment of saline oily water using membrane distillation, Desalination 566 (2023).
[9] M. Lou, S. Huang, X. Zhu, J. Chen, X. Fang, F. Li, Dual-polymers inserted graphene oxide membranes with enhanced anti-wetting and anti-scaling performance for membrane distillation, J. Membr. Sci. 697 (2024).
[10] M. Tang, L. Zheng, D. Hou, X. Jia, J. Wang, Microstructure design and construction of anti-wetting and anti-fouling multifunctional Janus membrane for robust membrane distillation, Chem. Eng. J. 430 (2022).
